# Gene expression correlates and mechanistic insights into electric organ discharge duration changes in mormyrid electric fish

**DOI:** 10.1242/jeb.249548

**Published:** 2025-06-04

**Authors:** Mauricio Losilla, Jason R. Gallant

**Affiliations:** ^1^ Department of Integrative Biology, Michigan State University, East Lansing, MI 48824, USA; ^2^ Graduate Program in Ecology, Evolution and Behavior, Michigan State University, East Lansing, MI 48824, USA

**Keywords:** Na^+^ channel, K^+^ channel, Electrocyte, Androgen hormones, Testosterone

## Abstract

Electric organ discharge (EOD) duration in African weakly electric fish (Mormyridae) is the most variable waveform component between species and the basis for distinguishing species-specific signals. EOD duration is thought to be influenced by morphological and physiological features of electrocytes (the cells that comprise the electric organ), but the mechanistic details are poorly understood. It has long been known that EOD duration is modulated by androgen hormones, affording an opportunity to identify gene expression correlates of EOD duration differences. We induced EOD elongation in the mormyrid *Brienomyrus brachyistius* by administering 17α-methyltestosterone (17αMT) to three treatment groups: control (no 17αMT exposure), T1day and T8day (samples taken 1 and 8 days after a single exposure to 17αMT, respectively). We then performed RNAseq, differential gene expression and functional enrichment analysis to detect gene expression changes during EOD duration change. Our analyses indicate 44 genes whose expression changed in tandem with EOD elongation and include genes responsible for actin filaments and microtubules, extracellular matrix organization and membrane lipid metabolism. Additionally, we found expression changes in one Na^+^ channel β-subunit, and five voltage-gated K^+^ channels. Together, these genes point toward specific cellular processes that contribute to morphological and physiological changes that contribute to EOD duration changes.

## Teaser

**Summary:** Androgen hormones drive gene expression changes that shape electrocyte morphology and ion channel activity, unlocking the molecular mechanisms behind electric signal duration elongation in mormyrid fish.

## INTRODUCTION

Despite little ecological differentiation, African weakly electric fish (Mormyridae) hold one of the highest rates of speciation among ray-finned fishes (Carlson et al., 2011; Rabosky et al., 2013). It is suspected that a crucial factor of their extraordinary diversification is divergence in their electric organ discharges (EODs). In *Paramormyrops*, EODs have evolved faster than size, morphology and trophic ecology (Arnegard et al., 2010a). These electric signals, crucial to electrolocation (Lissmann and Machin, 1958; von der Emde et al., 2008) and communication (Kramer, 1974; Möhres, 1957), are effective mechanisms of prezygotic isolation (Arnegard et al., 2006; Hopkins and Bass, 1981), and often are the most reliable method for identifying species (Arnegard et al., 2005; Bass, 1986; Hopkins, 1981).

Species-specific mormyrid EOD waveforms vary primarily in their complexity (number of phases), polarity (alterations to waveform shape caused by a large initial phase, P0) and duration (Hopkins, 1999a; Sullivan et al., 2002), the last of these being the most variable factor between species (Arnegard and Hopkins, 2003; Gallant et al., 2011) and the basis for recognition of species-specific signals (Arnegard et al., 2006; Hopkins and Bass, 1981). EOD duration differs up to 100× among species (Hopkins, 1999b), ranging from 0.2 to >15 ms (Lavoué et al., 2008); it is often sexually dimorphic (Bass and Hopkins, 1983; Hopkins, 1999b), with differences between male and female adult EODs often exacerbated during the rainy season breeding period (Hopkins and Bass, 1981).

The distinctive features of the EOD waveform are a consequence of the physiological, morphological and ultrastructural characteristics of the electrocytes (Carlson, 2002). Mormyrid EODs are produced by the synchronized discharge of the electrocytes, the constitutive cells of the electric organ, located in the caudal peduncle (Hopkins, 1986). The EOD from an individual electrocyte is composed of the sum of the action potentials (APs) from different parts of its excitable membrane. The two largest phases of a typical mormyrid EOD, called P1 and P2, are generated by the combined APs of the posterior and anterior faces of the electrocyte membrane (Bennett and Grundfest, 1961). These two membranes are thought to receive the depolarization signal simultaneously, but ions begin flowing through the posterior face first (Stoddard and Markham, 2008). The reasons for this delay could be physiological (ion channels) and/or due to greater capacitance of the more intricately folded anterior face; however, the majority of the two APs still overlap. Therefore, the resulting EOD is the net current of overlapping ingoing and outgoing ionic currents at the two faces of the membrane (reviewed in Dunlap et al., 2017; Markham, 2013; for a simplified figure of this model, see Stoddard and Markham, 2008). This model is thought to apply broadly to biphasic EODs from mormyrids and the Gymnotiformes (a diverse clade of independently evolved neotropical weakly electric fish). Biphasic EODs have only P1 and P2, but several mormyrids produce triphasic waveforms with an additional phase P0. Their electrocytes have stalks that penetrate the electrocyte (Pa configuration); P0 is produced when current flows through these penetrating stalks (for more details, see Bennett, 1971; Bennett and Grundfest, 1961; Szabo, 1961).

EOD duration is thought to be regulated by both morphological and physiological aspects. Electrocytes with a larger membrane surface area have been correlated with EODs of longer duration (Bennett, 1971; Paul et al., 2015), especially when the area increase is more prominent in the anterior face of the membrane (Bass et al., 1986; Freedman et al., 1989; Nguyen et al., 2020; Paul et al., 2015). Presumably, the larger area raises the capacitance of the membrane, and thus increases the time it takes to start depolarizing (Bass and Volman, 1987; Bass et al., 1986; Stoddard and Markham, 2008). If this delay is more pronounced in the anterior face of the membrane, the net effect should be to extend EOD duration. In addition, changes to the abundance or kinetics of the ion channels in the electrocyte membrane are expected to also modulate EOD duration. In gymnotiforms, it has been demonstrated that the main electrocyte ionic currents are generated by voltage-gated Na^+^ and K^+^ currents (e.g. Ferrari and Zakon, 1993; Shenkel and Sigworth, 1991) and it is broadly expected that the same holds true in mormyrid electrocytes, where both Na^+^ (Arnegard et al., 2010b; Zakon et al., 2006) and K^+^ (Nagel et al., 2017; Swapna et al., 2018) voltage-gated channels are expressed, and differential expression of ion channel genes has been linked with interspecific differences in EOD waveform (Lamanna et al., 2015; Losilla et al., 2020).

We recently performed an examination of differential gene expression among closely related members of the genus *Paramormyrops* (Losilla et al., 2020), where we identified expression correlates of EOD duration. Based on the results of this study, we were motivated to link observed patterns of differential gene expression between species to mechanisms underlying changes in EOD duration that occur within the lifetime of an individual. Previous studies have indicated that androgen hormones, including 17α-methyltestosterone (17αMT) increase EOD duration in several mormyrid species, an effect that mimics natural sex differences (Bass and Hopkins, 1983, 1985; Bass and Volman, 1987; Bass et al., 1986; Freedman et al., 1989; Herfeld and Moller, 1998). Although elongated EODs during the breeding season are a male character, androgen hormone treatment of female mormyrids elicits a male-like response in the duration of the EOD (Bass and Hopkins, 1983, 1985; Bass and Volman, 1987; Bass et al., 1986; Freedman et al., 1989; Herfeld and Moller, 1998). Androgen hormones provoke similar responses on the duration of male and female EODs in gymnotiforms (e.g. Dunlap and Zakon, 1998; Few and Zakon, 2001; Mills and Zakon, 1987; Silva, 2019).

Treatment with androgen hormones can provoke large changes in EOD duration under controlled circumstances, providing a hitherto unexplored opportunity to identify the expression correlates of EOD duration differences. In this study, we leveraged this paradigm to investigate the molecular underpinnings of changes in EOD duration in the mormyrid *Brienomyrus brachyistius*, a species where males greatly elongate their EOD under breeding conditions, influenced by their social status and in connection with androgen levels in the blood (Carlson et al., 2000). We experimentally applied 17αMT to *B. brachyistius* individuals over a 9 day experimental period and examined patterns of differential gene expression over the course of this treatment compared with control organisms. Unlike our previous study, which compared gene expression between species, this analysis focused on conspecific adult individuals in controlled environments, eliminating critical confounding factors such as phylogenetic divergence and additional EOD variation. Our analysis strongly supports known aspects of morphological and physiological bases of EOD duration, and for the first time identifies specific genes and broad cellular processes that alter morphological and physiological properties of electrocytes during seasonally plastic EOD changes.

## MATERIALS AND METHODS

### Study fish

Our study was performed on 20 adult (≥110 mm) *Brienomyrus brachyistius* (Gill 1862) purchased through the pet trade. Males were identified by the presence of an indentation in the anal fin, a common dimorphic feature in mormyrids. Sex was confirmed by gonad inspection, after which we believe that 19 fish were males and one was female.

### Experimental conditions

**Fig. 1.**
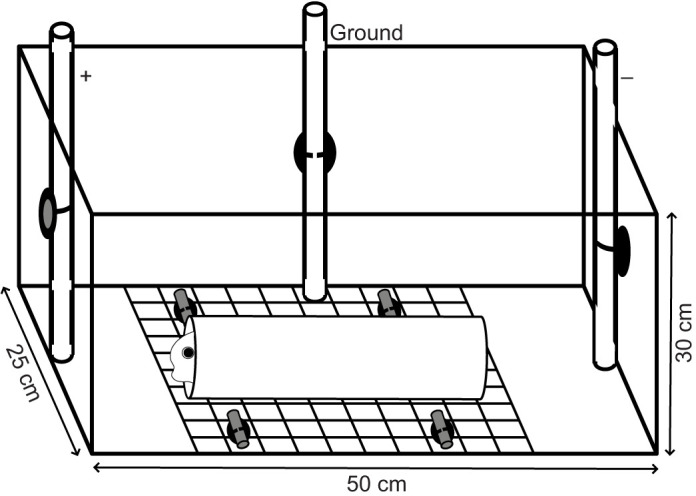
**Experimental fish tank configuration.** The housing arrangement was attached to the bottom of the tank, positioned in the middle. The fixed electrode locations (positive, negative and ground) are indicated.

Subjects were housed individually in 25 cm width×50 cm length×30 cm depth tanks filled with 30 l of water and equipped with one mechanical and biological filter. We kept the fish under 12 h light cycles, fed them blackworms daily, monitored levels of ammonia, nitrites and nitrates, and performed 30% water changes as needed during acclimation periods (no water changes were carried out after experimental treatments were applied). The following per tank water conditions were monitored daily or every other day and remained within the indicated limits: conductivity 500–570 µS cm^−1^, temperature 24–26°C, pH 7.0–7.5. Each tank was provided with a custom-built housing arrangement that served as a fish shelter but also facilitated measuring EODs with the fish at a stable location. These arrangements consisted of a PVC tube attached to an aquarium plastic egg crate via two zip ties; the egg crate was cut to the width of the tank and fixed to the bottom with four suction cups with hose clips, each with a short piece of PVC tube acting as a lock. The housing tube was centered at the bottom, oriented from the front to the back end of the tank (Fig. 1).

### EOD recordings and statistical analysis

For each subject, we recorded daily EOD waveforms (median 19, range 14–20) during acclimation and experimental periods. During recording sessions, electrodes were attached to suction cups with hose clips affixed to the center of the front (positive electrode), back (negative) and left (ground) sides of each tank (Fig. 1). The distance between the positive and negative electrodes was 48 cm. Signals were recorded with bipolar chloride-coated silver wire electrodes, amplified (bandwidth 0.0001–50 kHz, gain 20) with a bioamplifier (model BMA-200; CWE, Inc.), digitized using a multifunction DAQ device (model USB-6216 BNC; National Instruments Corp., Austin, TX, USA) and the custom recording software EODrecorder2 (settings: Terminal Configuration=Differential, sampling rate=250 kHz, voltage range=±5 V, npts=2048).

For each EOD waveform in each EOD recording, we corrected baseline offset by subtracting the average of the first and last 512 points from the waveform, then normalized by its peak-to-peak amplitude and aligned waveforms at their maximum head positive voltage (termed vP1). From here, individual EOD waveforms were averaged to minimize noise. All timing measurements were made relative to vP1 where time was arbitrarily assigned 0. The other key landmark is the minimum head positive voltage (termed vP2). We defined the start and end points of an EOD based on a threshold of 0.5% of its peak-to-peak amplitude. The start of an EOD was the first point of the first three consecutive points that exceeded the threshold. The last point was identified when the average voltage of the previous 11 points was larger than the negative threshold. EOD duration was calculated as the difference of these two values.

We calculated the following additional EOD waveform parameters: P1 duration, P2 duration, vP2/vP1, the maximum slope of P1 (sP1), P2 decay time constant (τ), and P1–P2 delay. P1 duration was calculated as the difference in time between EOD start and the zero-crossing between P1 and P2, and P2 duration was calculated as the difference in time between the same zero crossing and the EOD end. vP2/vP1 measures changes in the relative amplitude of P1 and P2. We calculated the derivative of each waveform to determine sP1, and we fitted an exponential decay function *y*=*a*×*e*^−τ*t*^+*b* to the voltages between P2 and EOD end point to determine τ. P1–P2 delay is the time elapsed between vP1 and vP2.

We tested for differences in EOD duration with a one-way ANOVA at (1) day 0 and (2) the last day of treatment (i.e. day 1 for T1day, day 8 for control and T8day; see description of experimental treatments below). Where significant results were detected, we examined differences in all pairwise treatment comparisons with the *post hoc* Tukey HSD test. We employed the same approach to detect differences in each of the additional EOD waveform parameters we calculated.

### Experimental treatments

Each subject was acclimated to the experimental setup for a minimum of 2 weeks before the start of a treatment. We deemed a fish acclimated when the coefficient of variation (CV) of EOD duration was<5% for five consecutive days. Experimental treatments were conducted in two back-to-back cycles, with 8 and 12 fish, respectively. For each cycle, treatment was applied on day 0, immediately after taking the EOD recordings for that day. Fish were assigned to one of three experimental treatments: two groups received 3 mg l^−1^ of 17αMT dissolved in 5 ml of 100% ethanol administered directly to the tank water and were allowed to survive 1 day (T1day; *n*=7) or 8 days (T8day; *n*=7) post-treatment. The third group (control; *n*=6) received 5 ml of 100% ethanol administered directly to the tank water. We monitored the fish and recorded EODs daily, until and including the assigned dissection date. Our experimental setup (fixed housing arrangement, EOD recordings taken in each fish's tank, and non-invasive 17αMT addition) was designed to minimize fish manipulation and disturbance. The study individuals did not undergo surgery or injections, and once placed in its experimental tank, a fish was not removed or otherwise handled until its dissection date. All procedures complied with federal and state regulations and were approved by Michigan State University's Office of Environmental Health and Safety, and Institutional Animal Care and Use Committee.

### Tissue dissections, RNA extraction and sequencing

After the final EOD recording session, subjects were euthanized with an overdose of MS-222 (0.7 g MS-222 in 2 l of fish tank water and 1.4 g of sodium bicarbonate). We skinned and dissected caudal peduncles and stored them in RNA-later (Ambion, Inc.), following the manufacturer's instructions, until processing. All RNA extraction, library preparation and sequencing were performed by Genewiz, LLC (South Plainfield, NJ, USA). Total RNA was extracted using Qiagen RNeasy Plus Universal mini kit following the manufacturer's instructions (Qiagen, Hilden, Germany). Extracted RNA samples were quantified using Qubit 2.0 Fluorometer (Life Technologies, Carlsbad, CA, USA) and RNA integrity was checked using Agilent TapeStation 4200 (Agilent Technologies, Palo Alto, CA, USA). RNA poly-A selected sequencing libraries were prepared using the NEBNext Ultra II RNA Library Prep Kit for Illumina according to the manufacturer's instructions (NEB, Ipswich, MA, USA). Briefly, mRNA was first enriched with oligo(dT) beads then fragmented for 15 min at 94°C. First strand and second strand cDNA were subsequently synthesized. cDNA fragments were end repaired and adenylated at the 3′ end, and universal adapters were ligated to the cDNA fragments, followed by index addition and library enrichment by limited-cycle PCR. The sequencing libraries were validated on the Agilent TapeStation and quantified using Qubit 2.0 Fluorometer as well as by quantitative PCR (qPCR; KAPA Biosystems, Wilmington, MA, USA). The sequencing libraries were clustered on flow cells. After clustering, the flow cells were loaded on to the Illumina HiSeq instrument (4000 or equivalent) according to the manufacturer's instructions. The samples were sequenced using a 2×150 bp paired end (PE) configuration. Image analysis and base calling were conducted by the HiSeq Control Software (HCS). Raw sequence data (.bcl files) generated from Illumina HiSeq was converted into fastq files and de-multiplexed using Illumina's bcl2fastq 2.17 software. One mismatch was allowed for index sequence identification.

### Read processing and data exploration

We inspected raw and processed reads with FastQC v.0.11.7 (Babraham Bioinformatics) and used Trimmomatic v.0.39 (Bolger et al., 2014) to remove library adaptors and low quality reads, and to filter small reads. The succeeding steps were executed using scripts included with Trinity v.2.11.0 (Grabherr et al., 2011; Haas et al., 2013). We aligned reads from each specimen to the predicted transcripts of the NCBI-annotated (release 100) *B. brachyistius* genome, with bowtie2 v.2.3.4.1 (Langmead and Salzberg, 2012). Expression quantification was estimated at the gene level using RSEM v.1.3.0 (Li and Dewey, 2011), followed by exploration of the data with a gene expression correlation matrix based on Euclidean distances and Pearson's correlation coefficient (for genes with read counts >10, Trinity's default parameters).

### Differential gene expression

We found no outliers for gene expression or EOD duration; hence, we analyzed samples lumped together in regard to sex and experimental cycle. We identified differentially expressed genes (DEGs) between experimental treatments in the three possible pairwise comparisons (control versus T8day, control versus T1day, T1day versus T8day) with edgeR v3.20.9 (Robinson and Oshlack, 2010) through a script provided with Trinity. For each comparison (contrast), we conservatively identified significant DEGs as those genes with a minimum expression fold change (FC) of 4 and a *P*-value <0.001 after FDR correction.

### Functional enrichment analysis

We employed the R package mitch v.1.8.0 (Kaspi and Ziemann, 2020) to detect sets of genes that exhibited joint upregulation or downregulation across our three contrasts. As inputs, mitch needs a gene set library and profiled expression data. The latter was the whole set (i.e. not filtered by FC or *P*-value thresholds) of edgeR differential expression results from each contrast. For the gene set library, we used a *Danio rerio* gmt file with gene ontology (GO) (Ashburner et al., 2000; Carbon et al., 2021) terms from the GO domain Biological Process as gene sets. To generate this file, we employed the script update_GO.sh from GeneSCF v.1.1-p3 (Subhash and Kanduri, 2016). As the gene identifiers in the gmt file are different from those of the edgeR results, mitch requires a third input file that relates these gene identifiers. To create this file, we first identified homologous proteins predicted from the *B. brachyistius* reference genome and those predicted from *D. rerio* (GRCz11) by blastp (BLAST+ v.2.11.0; Camacho et al., 2009). For each protein, the top hit (*e*-value ≤1*e*^−10^) was used for annotation. Then, we used mygene v1.32.0 (Wu et al., 2013; Xin et al., 2016) to match the *D. rerio* proteins to *D. rerio* genes.

We excluded gene sets with fewer than 20 genes from the mitch analysis (minsetsize option in mitch_calc function); and filtered the enrichment result to gene sets with false discovery rate (FDR)-corrected *P*<0.01 and the higher dimensional enrichment score (*S*) >0.1, to minimize false positives. To further simplify the analysis interpretation, we employed the tool Visualize from AmiGO 2 (Carbon et al., 2009) to build GO graphs with the enriched GO terms, and based on their hierarchy and connectivity, we manually grouped the GO terms into broad functional categories.

### DEGs of highest interest for EOD duration

We used two procedures to identify the most interesting genes in relation to EOD duration from the significant DEG detected by edgeR. First, we started with the gene sets in the broad categories of most interest from the previous step. We further filtered these gene sets to those which show an enrichment change that correlates with the observed changes in EOD duration (see code repo for details: https://github.com/msuefishlab/EODduration_geneExpression). We then compared the genes that make up each of these select gene sets with the significant DEG from each contrast, and highlighted the genes common to both sources. Our second procedure consisted of manually selecting additional significant DEGs from each contrast based on their annotation. We prioritized (i) genes from general functional themes suspected to affect EOD phenotype and (ii) genes whose expression patterns correlated with the observed changes in EOD duration (i.e. we did not prioritize genes found to be differentially expressed only in the control versus T1day comparison).

**Fig. 2.**
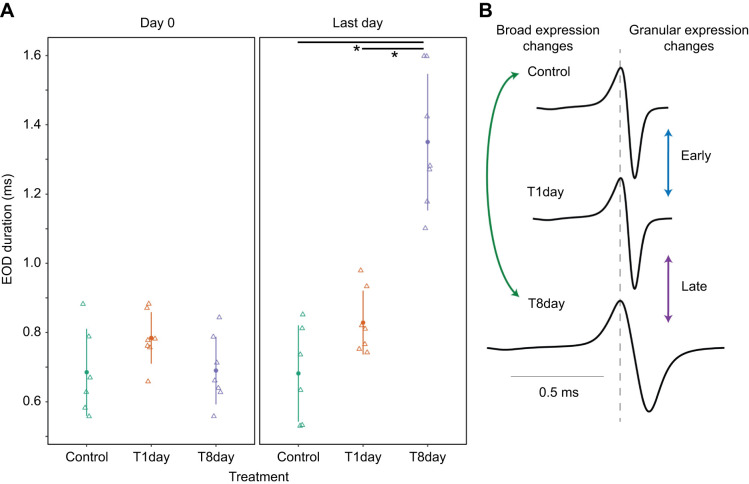
**Electric organ discharge duration in the experimental treatments and classification of gene expression changes.** (A) Electric organ discharge (EOD) duration at the onset (Day 0) and at the end (Last day) of the experiment for each of the treatments: T1day [17α-methyltestosterone (17αMT) treated, sampled on day 1], T8day (17αMT treated, sampled on day 8) and control (not treated with 17αMT, sampled on day 8). The filled circles and vertical lines represent the mean±s.d. EOD duration per treatment; open triangles are individual fish measurements. A small horizontal jitter was added to better visualize overlapping observations. Asterisks represents significant differences (*P*<0.001) between the treatments connected by the horizontal bar. (B) Each graph shows the traces of the averaged EODs from one representative fish per treatment, taken on their last day of recording. The arrows indicate the three pairwise treatment comparisons, and the gene expression changes detected in each comparison are classified as ‘broad’ or ‘granular’, based on each treatment's time of exposure to 17αMT. Granular changes are further divided into ‘early’ and ‘late’.

Our experimental design facilitates inferences about gene expression over the amount of time subjects were exposed to 17αMT (Fig. 2B). The comparison control versus T8day informs about broad changes in gene expression over the course of the experiment, whereas control versus T1day exposes early changes in gene expression, and T1day versus T8day uncovers late changes in gene expression. For example, a hypothetical gene set may show few ‘broad’ changes (control versus T8day), but important ‘granular’ changes in opposite directions before and after day 1 (control versus T1day, and T1day versus T8day). Note that we never observed conflicts between ‘broad’ and ‘granular’ expression patterns.

## RESULTS

### EOD duration responses to hormone treatment

There were no differences in the duration of the EOD between the treatments on day 0, immediately before treatment application (*P*=0.15, Fig. 2A). On the last day of treatment, shortly before tissue dissection, the duration of the EOD differed between the treatments (*P*<0.001); specifically, EOD duration increased in T8day fish compared with control (*P*<0.001) and T1day fish (*P*<0.001), but it did not differ between control and T1day fish (*P*=0.21) (Fig. 2A). Throughout the experiment, we observed stable mean EOD duration values during the last days of the acclimation period for all treatments and also during the experimental period for treatments control and T1day; however, there was a sharp increase in mean EOD duration for fish in the T8day treatment upon addition of 17αMT (Fig. S1). In addition to overall changes in duration, we also observed several significant changes in the additional EOD waveform parameters. Specifically, for treatment T8day compared with T1day and control, we detected significant increases in P1 duration, P2 duration, P1–P2 delay and vP1; and significant decreases in vP2, vP2/vP1 and τ (the last of these indicates a longer rate of P2 decay) (Table 1). The final EOD for a representative fish from each treatment is illustrated in Fig. 2B.

**Table 1.** Summary of electric organ discharge (EOD) measurements made prior to treatment (Day 0) and at the end of the experiment (Last day) for each treatment group

Day	Treatment	EOD duration (ms)	P1 duration (ms)	P2 duration (ms)	vP2/vP1	P1–P2 delay (ms)	τ	sP1	vP1	vP2
0	Control	0.68±0.13^a^	0.03±0.00^a,b^	0.04±0.00^a^	−1.44±0.20^a^	0.07±0.01^a,b^	31.06±4.70^a^	291.38±71.18^a^	0.41±0.03^a^	−0.59±0.03^a^
	T1day	0.78±0.07^a^	0.04±0.00^a^	0.04±0.01^a^	−1.46±0.20^a^	0.08±0.01^a^	26.88±5.54^a^	242.45±47.25^a^	0.41±0.03^a^	−0.59±0.03^a^
	T8day	0.69±0.09^a^	0.03±0.00^b^	0.04±0.00^a^	−1.54±0.21^a^	0.07±0.01^b^	32.04±4.08^a^	278.16±64.73^a^	0.40±0.03^a^	−0.60±0.03^a^
Last	Control	0.68±0.14^a^	0.04±0.01^a^	0.04±0.00^a^	−1.44±0.21^a^	0.07±0.01^a^	30.24±5.40^a^	293.72±77.33^a^	0.41±0.03^a^	−0.59±0.03^a^
	T1day	0.83±0.09^a^	0.04±0.01^a^	0.04±0.01^a^	−1.46±0.18^a^	0.08±0.01^a^	25.82±5.12^a^	237.71±45.81^a^	0.41±0.03^a^	−0.59±0.03^a^
	T8day	1.35±0.20^b^	0.07±0.01^b^	0.09±0.03^b^	−1.08±0.28^b^	0.16±0.04^b^	11.18±3.07^b^	227.10±57.83^a^	0.49±0.08^b^	−0.51±0.08^b^

We measured the EOD, P1 and P2 duration, the ratio of the P2/P1 maximum voltage (vP2/vP1), the delay between P1 and P2 (P1–P2 delay), the decay constant of P2 (τ), the maximum slope of P1 (sP1) and the maximum normalized amplitudes of P1 and P2 (vP1, vP2). The mean±s.d. is reported for each measurement. Significant differences are indicated with lowercase letters: groups with different letters within a measurement are significantly different (*P*<0.05).

### RNAseq data and DEGs

We explored the RNAseq data with a heatmap of pairwise correlations of gene expression across all 20 samples. Gene expression patterns across all specimens were highly correlated (Pearson's *r*>0.93), and correlation values were higher between fish from the same treatment (Fig. S2). At the level of treatments, these correlations indicate that fish from T1day and T8day have a more similar gene expression pattern than control fish.

We performed the three possible pairwise differential gene expression comparisons between treatments. They each revealed a similar number of expressed genes (∼20,750) and a small percentage (range 0.21–1.17%) of DEGs (FC >4, FDR-corrected *P*<0.001). Among the latter, there were always more genes upregulated in the treatment that entailed longer exposure to 17αMT (Table 2). Dataset 1 provides a tabular list of DEGs for every comparison, along with each gene's annotation, log_2_FC, *P*-value and per-fish trimmed mean of M (TMM) normalized expression values.

**Table 2.** All three possible pairwise differential gene expression comparisons with the number of genes expressed, the number differentially expressed and their breakdown by treatment

Comparison	No. genes expressed	No. genes differentially expressed (%)	No. upregulated genes	
			Treatment	Total
Control versus T8day	20,887	244 (1.17)	Control	96
			T8day	148
Control versus T1day	20,942	98 (0.47)	Control	34
			T1day	64
T1day versus T8day	20,401	43 (0.21)	T1day	13
			T8day	30

### Functional enrichment analysis

Next, we compared patterns of expression of pre-defined gene sets (GO terms, see Materials and Methods) across our three pairwise treatment comparisons (contrasts). The level of enrichment (upregulation or downregulation) of each gene set in each contrast is quantified by an enrichment score (*S*, range −1 to 1). These contrasts were constructed such that positive values of *S* represent upregulation with increased exposure to 17αMT, and negative values of *S* indicate downregulation with increased exposure to 17αMT.

**Fig. 3.**
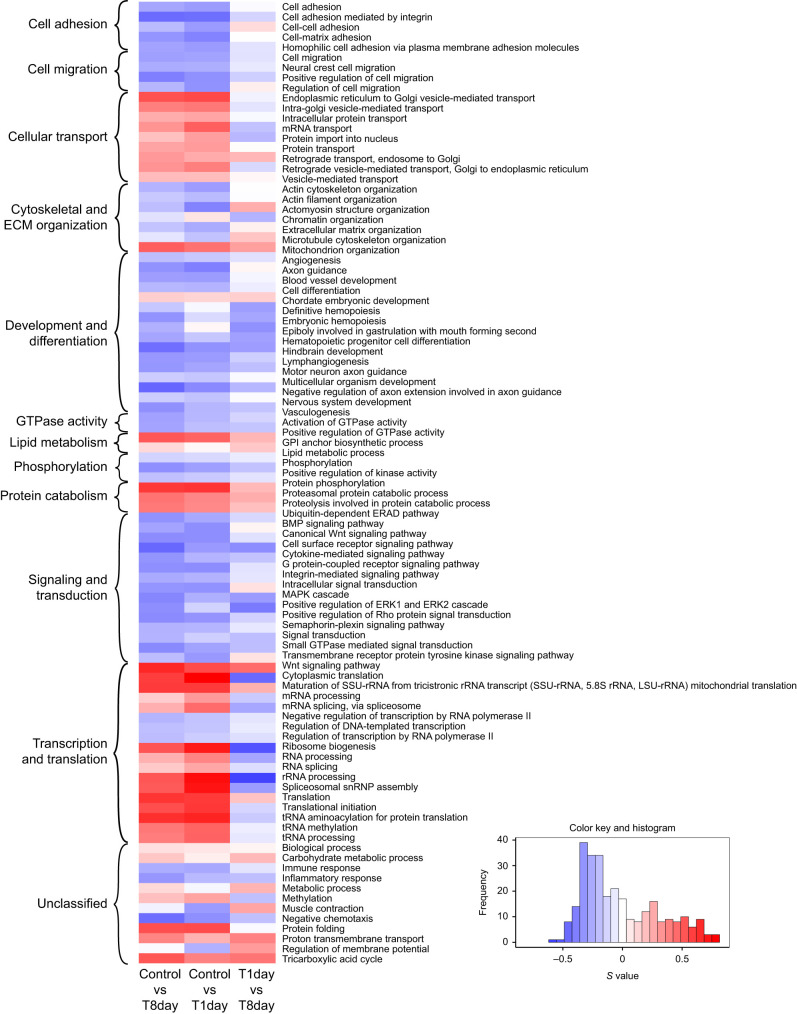
**Heatmap of mitch enrichment scores (*S*) for all significantly enriched gene sets in the three pairwise treatment contrasts studied.** *n*=96 gene sets, higher dimensional enrichment score >0.1, false discovery rate (FDR) *P*<0.01. Gene sets were further classified into broad categories (curly braces, left).

We detected 96 gene sets enriched across the three contrasts studied (Fig. 3; Dataset 2). To aid our interpretation, we further grouped these gene sets into 11 broad categories, with 12 gene sets unclassified because they were too general or too isolated in the GO graph (Fig. 3; Fig. S3).

Overall, the broad enrichment changes (control versus T8 day) per gene set were largely driven by early enrichment changes (control versus T1 day), whereas later changes in enrichment (T1day versus T8day) were generally more limited. For example, most gene sets in the categories ‘cell adhesion’, ‘cell migration’, ‘phosphorylation’ and ‘signaling and transduction’ were broadly downregulated, and the granular comparisons show that, in most of the constituent gene sets, downregulation happened quickly, while later changes were of smaller magnitude. A similar enrichment pattern, but with mostly upregulated genes instead, was generally observed in the gene sets across the categories ‘cellular transport’, ‘protein catabolism’ and ‘transcription and translation’ (Fig. 3).

Because of our overall motivation to examine genes that could contribute to anatomical and physiological changes that would affect EOD duration, we focused on the broad categories of most interest: ‘cell adhesion’, ‘cell migration’, ‘cytoskeletal and ECM organization’, ‘lipid metabolism’ and the unclassified gene sets ‘muscle contraction’ and ‘regulation of membrane potential’. We filtered their gene sets to those with an important change in enrichment magnitude and direction before and after day 1 (i.e. between the comparisons control versus T1day and T1day versus T8day), the time when EOD duration started to increase (Fig. S1). We call the resulting 19 gene sets ‘select gene sets’; they are: ‘actin cytoskeleton organization’, ‘actin filament organization’, ‘actomyosin structure organization’, ‘cell adhesion’, ‘cell adhesion mediated by integrin’, ‘cell migration’, ‘cell–cell adhesion’, ‘cell–matrix adhesion’, ‘chromatin organization’, ‘extracellular matrix organization’, ‘GPI anchor biosynthetic process’, ‘homophilic cell adhesion via plasma membrane adhesion molecules’, ‘lipid metabolic process’, ‘microtubule cytoskeleton organization’, ‘muscle contraction’, ‘neural crest cell migration’, ‘positive regulation of cell migration’, ‘regulation of cell migration’ and ‘regulation of membrane potential’. We list the genes that comprise the select gene sets in Dataset 2.

### DEGs of highest interest for EOD duration

We generated a list of genes that may directly influence EOD duration by (i) taking the genes common to at least one select gene set and the DEG from the pairwise comparisons, and (ii) from the latter, selecting additional genes that, based on their annotation, could contribute to EOD duration. As a guide, we used the general, functional themes suspected to affect EOD phenotype (Gallant et al., 2012, 2014; Lamanna et al., 2015; Losilla et al., 2020). For simplicity, we sorted these genes into the same themes we have used previously (Losilla et al., 2020): ‘extracellular matrix’, ‘cation homeostasis’, ‘lipid metabolism’ and ‘cytoskeletal & sarcomeric’. We removed from the resulting list those genes found differentially expressed only in the control versus T1 day comparison, to prioritize the genes with an expression pattern that resembles the observed changes in EOD phenotype. We also removed the gene *srd5a2* because its product converts testosterone to dihydrotestosterone. The outcome was a list of DEGs of highest interest for EOD duration. We provide the complete list (44 genes), along with functional annotations from UniProt (The UniProt Consortium, 2019), in Dataset 3. Select genes are mentioned in the Discussion and presented in Tables 3–5.

**Table 3.** **Select differentially expressed genes (DEGs) of highest interest likely to influence EOD duration from the general theme** ‘**cytoskeletal & sarcomeric**’

Entrez GeneID	Gene	Gene description	Gene set (enrichment analysis)	Contrast	FC
125707762	*actn1*	Actinin, alpha 1	Actin cytoskeleton organization	Control vs T8day	5.07
125707848	*actc1c*	Actin, alpha cardiac muscle		Control vs T8day	11.6
125707921	*myh6*	Myosin-6-like		Control vs T8day	216
125715397	*eml6*	Echinoderm microtubule-associated protein-like 6		Control vs T8day	4.4
125738327	*tubb2*	Tubulin beta-2A chain	Microtubule cytoskeleton organization	Control vs T8day	4.03
125738956	*acta1b*	Actin alpha 1, skeletal muscle b		Control vs T1day	−5.44
				T1day vs T8day	8.19
125740219	*xirp1*	Xin actin binding repeat containing 1	Actin cytoskeleton organization, actin filament organization	Control vs T1day	44.8
				Control vs T8day	50.9
125742816	*myo1d*	Myosin 1D	Actin filament organization	Control vs T1day	6.11
				Control vs T8day	6.04
125746486	*zgc:55461*	Beta-tubulin family protein	Microtubule cytoskeleton organization	T1day vs T8day	4.83
125748631	*cavin4b*	Caveolae associated protein 4b		Control vs T8day	18.5
125751135	*mid1ip1l*	Mid1-interacting protein 1-like		Control vs T8day	4.18

FC, fold change in gene expression. Positive values represent upregulation; negative values indicate downregulation, in the treatment that involved longer exposure to 17αMT.

**Table 4.** **Select DEGs of highest interest likely to influence EOD duration from the general themes** ‘**extracellular matrix**’ **and** ‘**lipid metabolism**’

Entrez GeneID	Gene	Gene description	Gene set (enrichment analysis)	Contrast	FC
125718703^a^	*si:dkey-61l1.4*	Collagen alpha-1(I) chain-like	Extracellular matrix organization	Control vs T8day	4.12
125720044^a^	*epdl2*	Ependymin-like 2	Cell–matrix adhesion	Control vs T8day	−4.57
125745631^a^	*thbs4b*	Thrombospondin 4b	Cell adhesion	Control vs T1day	5.97
				Control vs T8day	33.7
125751636^a^	*cdh11*	Cadherin 11, type 2, OB-cadherin (osteoblast)	Cell adhesion, cell–cell adhesion, homophilic cell adhesion via plasma membrane adhesion molecules	Control vs T8day	4.65
125709660^b^	*elovl7a*	ELOVL fatty acid elongase 7a	Lipid metabolic process	Control vs T1day	74.5
				Control vs T8day	105
125714471^b^	*selenoi*	Selenoprotein I		Control vs T1day	12.3
				Control vs T8day	18.8
125719038^b^	*fam126a*	Family with sequence similarity 126 member A		Control vs T1day	6.46
				Control vs T8day	5.8

FC, fold change in gene expression. Positive values represent upregulation; negative values indicate downregulation, in the treatment that involved longer exposure to 17αMT. ^a^Extracelluar matrix; ^b^lipid metabolism.

**Table 5.** **Select DEGs of highest interest likely to influence EOD duration from the general theme** ‘**cation homeostasis**’

Entrez GeneID	Gene	Gene description	Gene set (enrichment analysis)	Contrast	FC
125706737	*kcng4a*	Potassium voltage-gated channel, subfamily G, member 4a		Control vs T8day	7.82
125712673	*scn4b*	Sodium channel subunit beta-4-like		Control vs T8day	−9.23
125721870	*atp1b2b*	ATPase Na^+^/K^+^ transporting subunit beta 2b		Control vs T1day	8.52
				Control vs T8day	17.9
125722963	*gabrb3*	Gamma-aminobutyric acid type A receptor subunit beta3	Regulation of membrane potential	T1day vs T8day	7.86
				Control vs T8day	5.87
125738950	*kcnc2*	Potassium voltage-gated channel subfamily C member 2-like		Control vs T1day	−4.12
				T1day vs T8day	−4.99
				Control vs T8day	−20.9
125742760	*kcnc1*	Potassium voltage-gated channel subfamily C member 1-like		Control vs T8day	−10.4
125743020	*kcna7a1*	Potassium voltage-gated channel subfamily A member 7-like		Control vs T8day	−2.83
125750347	*kcna1*	Potassium voltage-gated channel subfamily A member 1		Control vs T8day	−16.1

FC, fold change in gene expression. Positive values represent upregulation; negative values indicate downregulation, in the treatment that involved longer exposure to 17αMT.

## DISCUSSION

In this study, we sought to identify gene expression patterns related to changes in EOD duration in samples from the same species, *Brienomyrus brachyistius*. In contrast with typical studies, a single focal species reduces confounding variability introduced by interspecific differences. We experimentally treated individually housed specimens with 17αMT for 1 or 8 days and compared patterns of gene expression with those of individuals treated with ethanol vehicle (control). By leveraging differential expression and functional enrichment analyses, we were able to systematically identify specific genes and broad functional themes that changed expression as the EOD elongated. It remains to be studied whether this experimental paradigm could elicit EOD duration changes in mormyrid species without sex or seasonal differences in their EODs, such as in the speciose genus *Campylomormyrus*.

### EOD duration and gene expression in the electric organ

By focusing only on adult *B. brachyistius*, our experimental design addressed confounding sources of variability such as phylogenetic divergence and age. Unlike other features of EODs such as complexity or polarity, duration can change relatively quickly depending on environmental conditions. Therefore, we housed fish individually, carefully monitored their acclimation, minimized disturbances, and elicited EOD elongation with a proven hormone manipulation. The resulting EODs were consistent through time and of similar duration across treatments before addition of 17αMT, and upon its application the recipient fish steadily increased their EODs (Fig. 2; Fig. S1). This response was not evident by day 1, but it increased progressively and became striking by day 8. This change was driven by a roughly equal, significant change in the duration of P1 and P2. These alterations in duration were accompanied by substantial changes in EOD shape by day 8 (Table 1): while EODs were initially asymmetric (P2 had a significantly larger amplitude than P1) they became more symmetric with 17αMT treatment (vP2/vP1), with the relative amplitude of vP2 decreasing. While the slope of P1 did not change, the delay between P1 and P2 increased, and there was a decrease in the time constant of the P2 decay (τ), indicating that P2 took longer to return to baseline in T8day EODs.

The critical EOD duration comparison between treatments is that of the last day of treatment (Fig. 2A, right) because the underlying recordings were taken shortly before tissue dissection. In other words, all the gene expression data derive from the electrocytes that generated these, and only these, EODs.

As a result of this design, we observed relatively low levels of differential expression between treatment groups (<1.17%; Table 2) and high correlation levels of gene expression across all 20 samples. Importantly, these correlation values were higher among samples of the same treatment (Fig. S2), implying that the differences in gene expression were driven by experimental manipulation. Notably, at treatment level, overall gene expression was more similar between T1day and T8day (Table 2, Fig. 3; Fig. S2), despite the 7 day difference between them, compared with a 1 day difference between control and T1day treatments. This strongly suggests that the majority of changes in gene expression had materialized by day 1, despite little change in EOD duration at this time (Fig. 2; Fig. S1). This emphasizes the importance of including the treatment T1day in our analysis: it provides granularity to the understanding of how 17αMT induces EOD elongation (Fig. 2B). In sum, given the robustness of our EOD duration and gene expression data, we expect that gene expression changes that underlie differences in EOD duration are amongst the DEGs we detected.

### Functional enrichment analysis

Functional enrichment analysis greatly simplifies the interpretation of the copious amount of data generated by omic technologies like RNA sequencing, by identifying previously defined groups of related genes that differ between the experimental treatments (García-Campos et al., 2015). We employed mitch, a multi-contrast enrichment analysis tool, to detect GO terms of biological processes jointly enriched across our three pairwise treatment contrasts (Fig. 3). To facilitate inferences about how these results may relate to our experimental manipulations and the ensuing changes in EOD duration, we further grouped these significant gene sets into broad categories (Fig. 3; Fig. S3). The per-category expression patterns uncovered a broad but coherent picture of sequential changes in gene expression that presumably start with 17αMT-induced modifications and likely culminate in a distinct set of gene expression changes that alter the EOD phenotype. We note that this analysis is limited by errors in the *D. rerio* gmt file, in the *B. brachyistius* genome annotation and in our procedure to match *D. rerio* and *B. brachyistius* genes.

#### Early changes in response to 17αMT

Our data demonstrate that exposure to 17αMT triggers a signal that is transduced to downstream effectors through cell signaling pathways. Anticipated as part of the cells' immediate response are changes in gene expression and increases in general protein-related processes, regardless of the ultimate, phenotypic effects induced. Accordingly, we broadly observed fast enrichment changes in the categories of ‘signaling and transduction’, ‘transcription and translation’, ‘cellular transport’, ‘protein catabolism’, and in the unclassified gene set ‘protein folding’ (Fig. 3, control versus T1day contrast). After day 1, the enrichment change in these categories was generally more subdued, suggesting that they mainly represent an immediate or general reaction to hormone exposure (Fig. 3, T1day versus T8day comparison). We propose that these proximate responses ultimately induce expression changes in the genes we deem of highest interest for EOD duration, and that among these genes are the effectors of the observed EOD elongation. Finally, the fast enrichment changes in the gene sets ‘mitochondrion organization’, ‘proton transmembrane transport’ and ‘tricarboxylic acid cycle’ likely reflect increases in energy consumption, presumably a consequence of the energetic costs of the initial cellular response.

#### Late changes in response to 17αMT

Energy consumption, as reflected in the mentioned gene sets, consistently displayed strong upregulation throughout the experiment. Suitably, longer EODs are predicted to be metabolically more costly (Hopkins, 1999b), and this has been corroborated in the gymnotiform *Brachyhypopomus gauderio* (Salazar and Stoddard, 2008). Furthermore, genes set in the categories ‘cytoskeletal and ECM organization’, ‘lipid metabolism’ and the unclassified gene set ‘muscle contraction’ generally showed marked enrichment changes after day 1 (Fig. 3), and they embody several of the major features thought to be responsible for EOD variation. The genes that underlie these categories could alter the duration of the EOD by inducing changes in the surface area of the electrocyte membrane, a characteristic that has been correlated with longer EODs in descriptive (Bennett, 1971; Nguyen et al., 2020; Paul et al., 2015) and 17αMT-mediated experimental work (Bass and Volman, 1987; Bass et al., 1986; Freedman et al., 1989). In addition, these genes may regulate electrocyte interactions with the extracellular matrix (ECM) and the connective tissue sheath, which may contribute to EOD phenotype (Losilla et al., 2020). Noteworthy, signal transduction through Rho GTPases (Fig. 3) is a key regulatory mechanism of the actin cytoskeleton, membrane protrusions and adhesion complexes (Hall, 1998).

Finally, the gene set ‘regulation of membrane potential’ also exhibited a strong enrichment change after day 1. This gene set falls into our functional theme ‘cation homeostasis’, the remaining theme expected to influence EOD variation. Genes in this theme could alter the duration of the EOD by regulating plasma membrane ion channels, as has been demonstrated in gymnotiforms (reviewed in Dunlap et al., 2017; Markham, 2013), and explored in mormyrids (Lamanna et al., 2015; Nagel et al., 2017; Swapna et al., 2018). Although our analysis only recovered one gene from this gene set (*gabrb3*, a ligand-gated chloride channel and part of the GABA receptor; Table 5), we detected voltage-gated ion channel genes differentially expressed in our contrasts and included them in our list of genes of highest interest.

### DEGs of highest interest for EOD duration

We refined our list of candidate genes most likely to modulate EOD duration by (1) taking the genes common to the differential gene expression analysis and to the select gene sets from the functional enrichment analysis; and, given the limitations of the functional enrichment procedure, (2) by further selecting DEGs that align with previous knowledge. One bias of the latter is our inevitably incomplete understanding of the functional effects of every gene. Readers curious about the remaining DEGs can find them in Dataset 1. Unless otherwise indicated, gene descriptions come from UniProt.

#### Genes that affect electrocyte morphology

The anterior and posterior faces of the electrocyte plasma membrane often increase their surface area by evaginations and invaginations of the membrane (Schwartz et al., 1975). These projections, depending on their characteristics, are referred to in the literature as papillae, tubules, calveoli or canaliculi. The larger membrane projections are supported by the cytoskeleton (Korniienko et al., 2021). Increases in the membrane surface area are associated with longer EODs (Bennett, 1971; Paul et al., 2015), mainly through projections on the anterior face (Bass et al., 1986; Freedman et al., 1989; Nguyen et al., 2020; Paul et al., 2015). These increases in membrane surface area may increase membrane capacitance that thus delays spike initiation and membrane repolarization (Bass and Volman, 1987; Bass et al., 1986). Our analysis of additional EOD waveform parameters shows both a significantly increased delay between P1 and P2, and a significantly decreased τ in T8day fish, both consistent with altered capacitance of the anterior electrocyte face. Given this framework, we anticipate ‘cytoskeletal & sarcomeric’ genes involved in longer EODs to be mostly upregulated in the treatment T8day, and indeed genes from this theme display this expression pattern. For example, we detected strong increases in the actin or actin-related genes: *actn1*, *actc1c*, *myh6*, *acta1b*, *xirp1* and *myo1d*; and in the microtubule-related genes: *eml6*, *tubb2*, *zgc:55461*, *cavin4b* and *mid1ip1l* (Table 3). Recent work on *Campylomormyrus* highlighted two actin-related genes whose electric organ expression decreased in species with longer EODs (Cheng et al., 2024). Furthermore, we expect that this increase in surface area also requires rearrangements in the phospholipid bilayer and in the electrocyte's interactions with the ECM and the connective tissue sheath. Previous research supports that these themes are related to variation in EOD phenotypes (lipid metabolism: Lamanna et al., 2015; Losilla et al., 2020; ECM: Losilla et al., 2020). In fish with longer EODs, we identified increased gene expression of *elovl7a* and *selenoi*, two genes that participate in the production of membrane lipids; and of the gene *fam126a*, which plays a key role in certain cell types with expanded plasma membrane (Table 4). Likewise, the same expression pattern was observed for a collagen gene (*si:dkey-61l1.4*) and for several adhesion genes, including *thbs4b* and *cdh11*, whereas we found reduced expression of *epdl2* in these fish (Table 4). In *Paramormyrops*, we have previously identified *epdl2*, whose product may participate in cell–matrix adhesion, as a gene that could affect the EOD waveform (Losilla et al., 2020) and that underwent duplications and functional specialization (Losilla and Gallant, 2023).

#### Genes that affect electrocyte physiology

Another mechanism to change EOD duration is alteration of electrocyte ionic currents, which can be achieved by modifying the functional properties or levels of expression of ion channels. In gymnotiforms, ionic currents are largely driven by voltage-gated Na^+^ and K^+^ channels (e.g. Ferrari and Zakon, 1993; Shenkel and Sigworth, 1991), and their modulation via androgen hormones affects EOD duration (e.g. Ferrari et al., 1995; Zakon et al., 1991). To the best of our knowledge, technical difficulties have hindered recordings of mormyrid electrocyte ionic currents, yet significant convergent evolution is expected. It has been shown that both Na^+^ (Arnegard et al., 2010b; Zakon et al., 2006) and K^+^ (Nagel et al., 2017; Swapna et al., 2018) voltage-gated channels are expressed in mormyrid electrocytes, and differential expression of ion channel genes has been linked with EOD variation (Lamanna et al., 2015; Losilla et al., 2020).

Our analysis of additional EOD waveform parameters showed a significant relative increase in the amplitude of vP1 with no change in the rising slope of P1 (sP1). From extracellular recordings of EODs, it is impossible to directly determine the decay rate of P1; however, we can infer that the decay rate was longer in treatment T8day, from the significant delay of P2 relative to P1, as well as the increased duration of P1. Given that both P1 and P2 duration increased, we hypothesize that ion channel expression changes likely occurred in both anterior and posterior electrocyte membranes. Furthermore, because the duration increase was more pronounced for P2, the largest change in ion flow comes from the potassium ions exiting through the anterior membrane. We note that this is only one potential interpretation: in order to tie changes in ionic currents to electrocyte regions it would be necessary to obtain additional data from physiological recordings from the electrocyte faces, or *in situ* hybridization of candidate gene products.

First, we identified a gene that codes for a Na^+^ channel β-subunit, *scn4b*, downregulated in samples with longer EODs (Table 5). This mirrors results observed in the gymnotiform *Sternopygus macrurus*, where exposure to an androgen hormone elongated the EOD and downregulated the expression of a Na^+^ channel β1-subunit that probably speeds inactivation rates of the α-subunits (Liu et al., 2007). Given this mode of action, suppression of the β-subunit would likely extend the electrocyte Na^+^ currents and the EOD.

Second, we observed a striking coordinated regulation of at least five voltage-gated K^+^ channels genes: *kcna1*, *kcnc1*, *kcnc2*, *kcng4a* (Table 5) and *kcna7a1* (Entrez GeneID 125743020, potassium voltage-gated channel subfamily A member 7-like, see below). Based on the model of EOD production (e.g. Dunlap et al., 2017; Markham, 2013; Stoddard and Markham, 2008), APs from each electrocyte face, and therefore the resulting EOD, would elongate if K^+^ efflux is delayed or its rate reduced. Allowing K^+^ ions out of the cell is the canonical function of delayed-rectifier K^+^ channels; and two of these, Kv1.1a and Kv1.2a, are less expressed in longer EODs in *S. macrurus*, a response that can be elicited by androgen hormones (Few and Zakon, 2007). We detected four differentially expressed delayed-rectifier K^+^ channels, and in agreement with expectations, all of them were strongly downregulated in fish with longer EODs (T8day treatment): *kcna1* (Kv1.1), *kcna7a1* (Kv1.7), *kcnc1* (Kv3.1) and *kcnc2* (Kv3.2). Interestingly, Kv3.2 channels show adaptations for fast repolarization in neurons (Lien and Jonas, 2003; Rudy and McBain, 2001), positioning *kcnc1* and *kcnc2* as candidates for electrocyte specialization in EODs of short duration. We also identified gene *kcng4a* (Kv6.4), but unlike the previous voltage-gated K^+^ channel genes, this one was found upregulated in longer EODs. The protein coded by this gene is classified as a modifier/silencer channel that modulates the delayed-rectifier K^+^ channel *kcnb1* (Kv2.1). The addition of Kv6.4 causes a shift towards more negative potentials in the voltage dependence of steady-state inactivation (Bocksteins et al., 2012; Lee et al., 2020). If this shift influences EODs, the anticipated effect would be to close the channel faster (at more negative potentials), thus reducing potassium efflux, extending the AP and the ensuing EOD. In comparison, a different set of K^+^ channel genes, one voltage gated, one outward rectifier and one inward rectifier, have been linked to EOD duration across *Campylomormyrus* species (Cheng et al., 2024).

Finally, we note that *kcna7a* was not detected in our differential expression analysis; however, it has been reported that this gene codes for mormyrid-exclusive amino acid substitutions that enable ultra-brief EODs and that it is expressed at much higher levels than other voltage-gated K^+^ channels in mormyrid electrocytes (Swapna et al., 2018). This prompted us to take a closer look at this gene. We found four *kcna7a* genes in the *B. brachyistius* genome assembly, but we suspect that they represent two *kcna7a* paralogs and an assembly error. Interestingly, a comparable tandem duplication was recently discovered in *C. compressirostris* (Cheng et al., 2023), thus raising the possibility that this duplication is widespread among mormyrids. We call the *B. brachyistius* paralogs *kcna7a1* (Entrez GeneID 125743020, and the spuriously assembled 125742764) and *kcna7a2* (Entrez GeneID 125743019, and the spuriously assembled 125742763). We conclude that *kcna7a2* is the *kcna7a* gene studied by Swapna et al. (2018), because we found it was expressed at much higher levels than the other voltage-gated K^+^ channels. This gene's expression did not significantly change in our treatments. However, we found the less expressed paralog *kcna7a1* downregulated in T8day fish compared with control fish (FC=−2.83, FDR-corrected *P*<0.001). Given this, we include this gene in our discussion and suggest that the significance threshold for differential expression of FC=4 may be too stringent for ion channel genes.

Following our findings, we propose that, in *B. brachyistius* EOD, Kv1.7a2 is the K^+^ channel that carries the most K^+^ out of the electrocytes, yet K^+^ efflux during EOD elongation is modulated, at least in part, through the concerted regulation of several, less expressed, voltage-gated K^+^ channels. However, because *scn4b* is the only voltage-gated Na^+^ channel gene we detected, we propose that its regulation is the main mechanism of modulating EOD duration that involves Na^+^ flow in this species.

### Conclusions

We believe that this work represents the best controlled effort to date to study gene expression changes that correlate with EOD changes, particularly EOD duration. Our results provide insights into the stepwise cellular process induced by androgen hormones that leads to EOD elongation. A fast, coordinated response, mediated through multiple signaling pathways, activates gene expression, protein metabolism and processes that increase energy availability. The downstream result is changes in gene expression that modify the morphological and physiological properties of electrocytes in ways that align perfectly with theoretical expectations on how these changes could increase EOD duration. Increases in electrocyte membrane surface area are associated with longer EODs, and we detected a concordant upregulation of genes that participate in plasma membrane expansion. Importantly, our results deepen our understanding of how an increase in membrane area is accomplished, by pointing out that these morphological changes likely occur in tandem with changes to electrocyte interactions with the ECM and to the supporting cytoskeleton, including increases in specific actin and tubulin proteins, two of its essential building blocks. Similarly, our results also support that changes to the physiological properties of the electrocyte, mediated by expression changes of ion channels, influence EOD duration. They implicate changes to ionic currents that agree with current theories of EOD function and are novel examples of convergence between gymnotiforms and mormyrids. These are the downregulation in longer EODs of a Na^+^ channel β-subunit and delayed-rectifier K^+^ channels. We found multiple voltage-gated K^+^ channels, mostly but not exclusively delayed-rectifiers, consistently modulated in accordance with expectations of how they would influence EOD duration. Interestingly, these do not include *kcna7a2*, the most expressed voltage-gated K^+^ channel in electrocytes. Our results not only support and deepen current ideas of how mormyrids module EOD duration but also provide a list of specific genes likely to mediate this regulation. Given the central role of EOD duration in EOD diversification, and the importance of the EOD in species divergence, these genes may be significant participants in the speciation process amid African weakly electric fish.

## Supplementary Material

10.1242/jexbio.249548_sup1 Supplementary information

Dataset 1. DEG detected in each of the three pairwise DGE comparisons. Positive values under logFC indicate genes upregulated in the treatment under sampleA, whereas negative values correspond to genes upregulated in the treatment under sampleB. Values under each sample are TMM-normalized expression values. Significance threshold was abs(log2 FC) > 2 and FDR < 0.001.

Dataset 2. Statistical details about the 96 significantly enriched gene sets and the genes that comprise the 19 select gene sets. All values generated by the mitch package.

Dataset 3. DEG of highest interest for EOD duration. Positive values under logFC indicate genes upregulated in the treatment under sampleA, whereas negative values correspond to genes upregulated in the treatment under sampleB.
