# Supplementary material for: Gene expression correlates and mechanistic insights into electric organ discharge duration changes in mormyrid electric fish
Source: J Exp Biol. 2025 Jun 4;228(11):jeb249548. doi: 10.1242/jeb.249548 (PMC12188244; doi:10.1242/jeb.249548)
Supplement: Supplementary information [file jexbio-228-249548-s1.pdf]

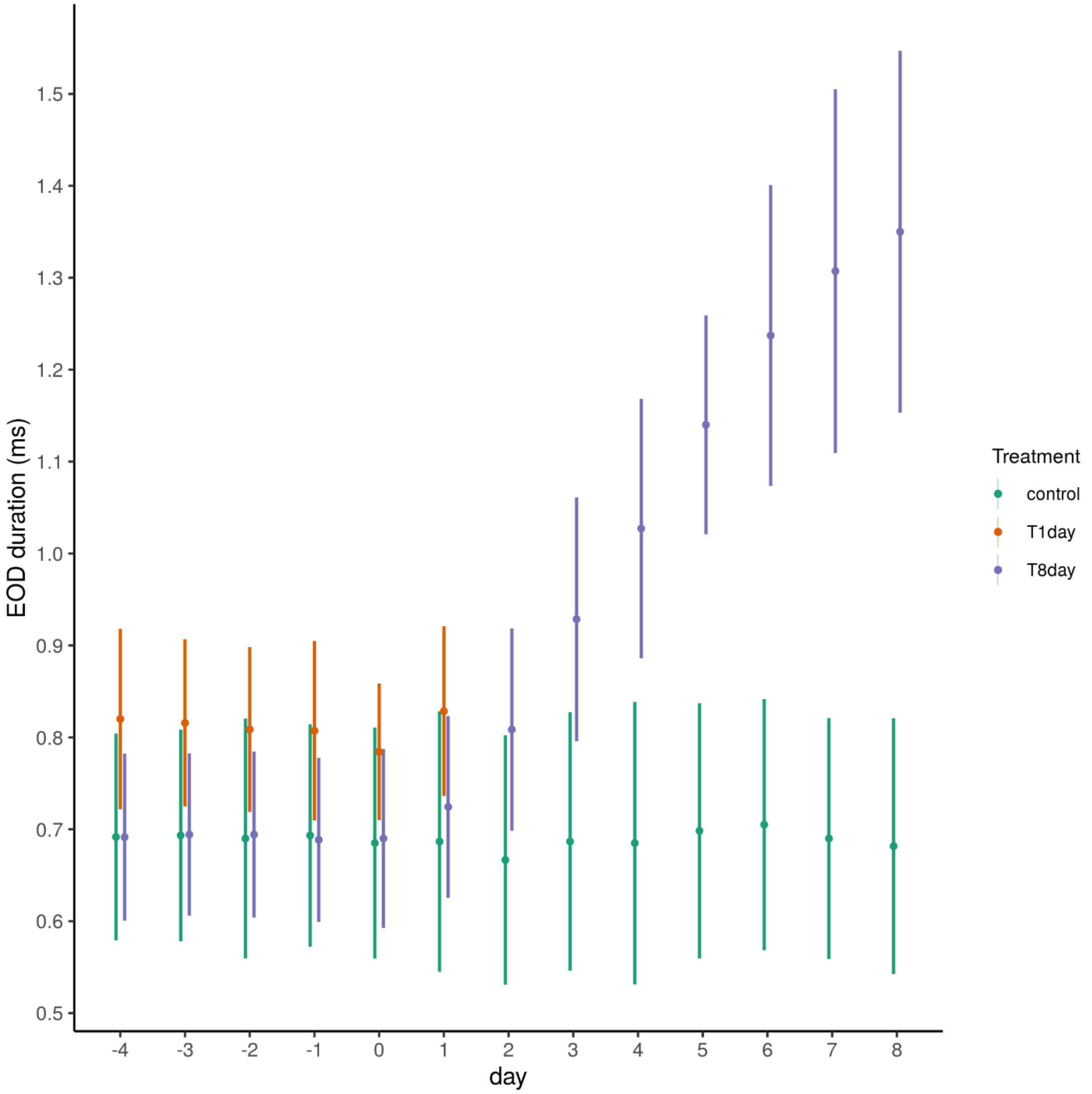


**Fig. S1.** EOD duration per treatment throughout the experiment. Each colored circle and its vertical lines represent the mean EOD duration ± s.d. per treatment and day. A small horizontal jitter was added to better visualize overlapping values. Days -4 to 0 are part of the acclimation period, treatment-specific manipulations were performed on Day 0 after taking EOD recordings.

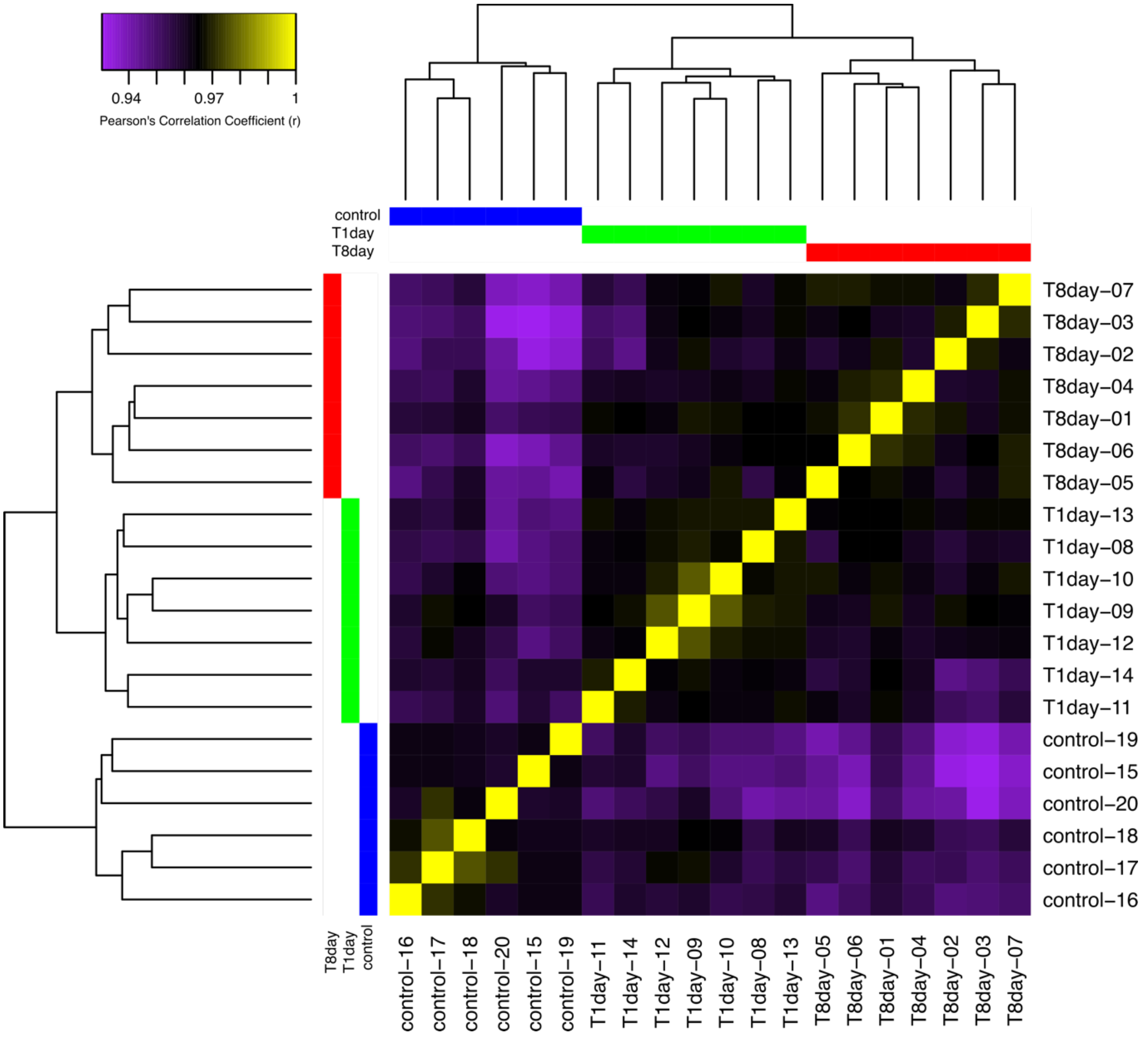


**Fig. S2.** Heatmap of sample by sample correlations in gene expression, and the inferred relationships among treatments from these expression correlation values.

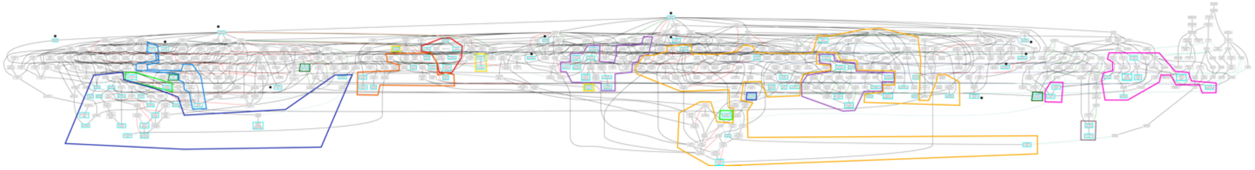

**Fig. S3.** Gene Ontology graph with the 96 enriched GO terms (cyan boxes) and their grouping into 11 broad categories (outlined by the colored shapes). 12 GO terms (black stars) remained unclassified because they were too general or too isolated.

Journal of Experimental Biology • Supplementary information

**Dataset 1.** DEG detected in each of the three pairwise DGE comparisons. Positive values under logFC indicate genes upregulated in the treatment under sampleA, whereas negative values correspond to genes upregulated in the treatment under sampleB. Values under each sample are TMM-normalized expression values. Significance threshold was abs($\log_2$ FC) > 2 and FDR < 0.001.

Available for download at
https://journals.biologists.com/jeb/article-lookup/doi/10.1242/jeb.249548#supplementary-data

**Dataset 2.** Statistical details about the 96 significantly enriched gene sets and the genes that comprise the 19 select gene sets. All values generated by the mitch package.

Available for download at
https://journals.biologists.com/jeb/article-lookup/doi/10.1242/jeb.249548#supplementary-data

**Dataset 3.** DEG of highest interest for EOD duration. Positive values under logFC indicate genes upregulated in the treatment under sampleA, whereas negative values correspond to genes upregulated in the treatment under sampleB.

Available for download at
https://journals.biologists.com/jeb/article-lookup/doi/10.1242/jeb.249548#supplementary-data
